# Targeting NRF2 for Improved Skin Barrier Function and Photoprotection: Focus on the Achiote-Derived Apocarotenoid Bixin

**DOI:** 10.3390/nu9121371

**Published:** 2017-12-18

**Authors:** Montserrat Rojo de la Vega, Andrea Krajisnik, Donna D. Zhang, Georg T. Wondrak

**Affiliations:** Department of Pharmacology and Toxicology, College of Pharmacy & Arizona Cancer Center, University of Arizona, Tucson, AZ 85724, USA; emrvg@email.arizona.edu (M.R.d.l.V.); akrajisnik@email.arizona.edu (A.K.); dzhang@pharmacy.arizona.edu (D.D.Z.)

**Keywords:** skin photodamage, skin barrier function, solar ultraviolet (UV), NRF2, PPARα, bixin, achiote

## Abstract

The transcription factor NRF2 (nuclear factor-E2-related factor 2) orchestrates major cellular defense mechanisms including phase-II detoxification, inflammatory signaling, DNA repair, and antioxidant response. Recent studies strongly suggest a protective role of NRF2-mediated gene expression in the suppression of cutaneous photodamage induced by solar UV (ultraviolet) radiation. The apocarotenoid bixin, a Food and Drug Administration (FDA)-approved natural food colorant (referred to as ‘annatto’) originates from the seeds of the achiote tree native to tropical America, consumed by humans since ancient times. Use of achiote preparations for skin protection against environmental insult and for enhanced wound healing has long been documented. We have recently reported that (i) bixin is a potent canonical activator of the NRF2-dependent cytoprotective response in human skin keratinocytes; that (ii) systemic administration of bixin activates NRF2 with protective effects against solar UV-induced skin damage; and that (iii) bixin-induced suppression of photodamage is observable in *Nrf2*^+/+^ but not in *Nrf2*^−/−^ SKH-1 mice confirming the NRF2-dependence of bixin-induced antioxidant and anti-inflammatory effects. In addition, bixin displays molecular activities as sacrificial antioxidant, excited state quencher, PPAR (peroxisome proliferator-activated receptor) α/γ agonist, and TLR (Toll-like receptor) 4/NFκB (nuclear factor kappa-light-chain-enhancer of activated B cells) antagonist, all of which might be relevant to the enhancement of skin barrier function and environmental stress protection. Potential skin photoprotection and photochemoprevention benefits provided by topical application or dietary consumption of this ethno-pharmacologically validated phytochemical originating from the Americas deserves further preclinical and clinical examination.

## 1. Introduction: Solar Radiation, Photodamage, Photoaging, and Skin Photocarcinogenesis

Exposure to solar ultraviolet (UV) radiation is a causative factor in acute skin photodamage, chronic photoaging, and photocarcinogenesis [1,2,3,4]. More recently, a causative role of solar photons in the visible and infrared spectral range contributing to skin photodamage has been substantiated [5,6,7,8]. Moreover, cutaneous exposure to other environmental stressors including combustion pollutants, heavy metals, metalloids, and ozone has been shown to contribute to skin damage and carcinogenesis. Remarkably, nonmelanoma skin cancer (NMSC; also referred to as keratinocyte cancers (KC)) is the most common malignancy in the United States, and skin cancer incidence is increasing rapidly, presenting a public health burden of considerable magnitude [9]. Even though sunscreen-based photoprotection is an effective component of a sun-safe strategy to reduce cumulative lifetime exposure to UV light, much effort has been directed towards the development of more effective molecular strategies acting through mechanisms different from (or synergistic with) photon absorption [9,10,11,12,13,14].

## 2. NRF2: A Master Regulator of Skin Barrier Function, Cellular Defense Mechanisms against Environmental Stress, and Solar Radiation Response

The redox-sensitive transcription factor NRF2 (nuclear factor-E2-related factor 2) orchestrates major cellular defense mechanisms including phase-II detoxification, inflammatory signaling, DNA repair, and antioxidant response, and recent experimental evidence supports an important role of NRF2 in skin barrier function. NRF2 has therefore emerged as a promising molecular target for the pharmacological prevention of human pathologies resulting from exposure to environmental toxicants including solar UV-induced damage and carcinogenesis [15,16,17,18]. Moreover, the potential of NRF2 for modulation of skin chronological and photodamage-associated aging has attracted considerable attention [9,19,20].

## 3. NRF2: Molecular Biology and Pharmacological Modulation

**Figure 1:**
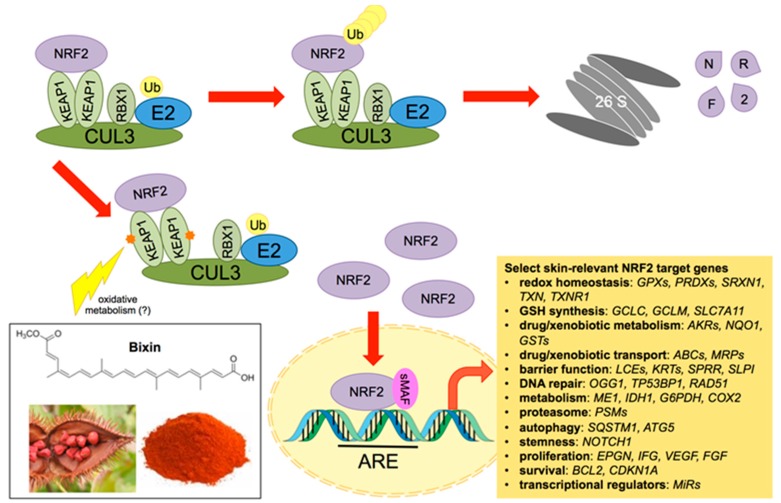
The nuclear factor-E2-related factor 2 (NRF2) pathway with a focus on skin barrier function and environmental stress protection. The transcription factor NRF2 binds to Kelch-ECH associated protein 1 (KEAP1), the substrate adaptor protein for the cullin 3-RING box protein 1 (CUL3-RBX1) E3 ubiquitin ligase complex. Under basal conditions, NRF2 is ubiquitylated and degraded by the 26S proteasome. Upon modification of reactive cysteines in KEAP1 by reactive oxygen species (ROS) and electrophiles (including bixin), NRF2 is no longer ubiquitylated. This allows for newly synthesized NRF2 to accumulate, translocate to the nucleus, and activate the transcription of antioxidant response element (ARE)-containing target genes by dimerizing with small MAF (sMAF) proteins. Select skin-relevant NRF2 target genes are displayed according to the cellular function they perform. *GPXs*, glutathione peroxidases; *PRDXs*, peroxiredoxins; *SRXN1*, sulfiredoxin 1; *TXN*, thioredoxin; *TXNR1*, thioredoxin reductase 1; *GCLC*, glutamate cysteine ligase, catalytic subunit; *GCLM*, glutamate cysteine ligase, modifier subunit; *SLC7A11*, glutamate/cystine antiporter (xCT); *AKRs*, aldoketoreductases; *NQO1*, NAD(P)H:quinone oxidoreductase 1; *GSTs*, glutathione S-transferases; *ABCs*, ATP-binding cassette family proteins; *MRPs*, multidrug resistance-associated proteins; *LCEs*, late cornified envelope family members; *KRTs*, keratins; *SPRR*, small proline rich proteins; *OGG1*, 8-oxo-guanine glycosylase; *TP53BP1*, p53 binding protein 1; *RAD51*, DNA repair protein RAD51 homolog 1; *ME1*, malic enzyme; *IDH1*, isocitrate dehydrogenase 1; *G6PDH*, glucose-6-phosphate dehydrogenase; *COX2*, cytochrome c oxidase subunit 2; *PSM*, proteasome subunit proteins; *SQSTM1*, sequestosome 1 (p62); *ATG5*, autophagy-related gene 5; *NOTCH1*, Notch homolog 1, translocation-associated; *EPGN*, epigen; *IGF*, insulin-like growth factor; *VEGF*, vascular endothelial growth factor; *FGF*, fibroblast growth factor; *BCL2*, B cell lymphoma 2; *CDKN1A*, cyclin dependent kinase inhibitor 1A (p21); *MiR*, microRNAs.

NRF2 is ubiquitously expressed in all tissues, including the skin, but its protein levels and consequently its activity are tightly regulated (Figure 1). Under basal (homeostatic) conditions, NRF2 resides in the cytosol, where it binds to its negative regulator Kelch-ECH associated protein 1 (KEAP1), a substrate adaptor for a cullin 3-RING box protein 1 (CUL3-RBX1) E3 ubiquitin ligase complex [21]. Thus, NRF2 is ubiquitylated and degraded by the 26 S proteasome [22]. However, upon exposure to reactive oxygen species (ROS) or to electrophilic compounds, key sensor cysteine residues in KEAP1 (cysteine 151 in particular) are chemically modified, causing a conformational change in KEAP1 that prevents degradation of NRF2, which remains complexed to KEAP1 [23,24]. This allows newly synthesized NRF2 to accumulate and translocate to the nucleus, where it heterodimerizes with small MAF (musculoaponeurotic fibrosarcoma) proteins and binds to the antioxidant response elements (AREs) in the regulatory regions of its downstream genes [25]. This mode of canonical NRF2 regulation has been extensively studied in the context of skin protection and pathogenesis. In addition, other modes of NRF2 regulation, such as the p62-dependent non-canonical pathway that activates NRF2 in an autophagy-dependent manner [26,27] or the GSK3-βTrCP (glycogen synthase kinase 3/β-transducin repeat containing protein) degradation pathway [28,29], have been described. However, the involvement of these other modes of NRF2 regulation in skin barrier function and environmental stress protection remain to be determined.

Many natural chemopreventive compounds that have antioxidant properties exert their cytoprotective function through NRF2 activation. Classic examples of NRF2 inducers are sulforaphane (from cruciferous vegetables) [16], curcumin (from *Curcuma longa*) [30], cinnamaldehyde (from cinnamon) [31,32], and tanshinones (from *Salvia miltiorrhiza*) [33], among many others. These compounds are promiscuous electrophilic molecules that also react with cysteine 151 of KEAP1, induce NRF2, and confer protection against a number of chemical insults or radiation damage (including UV) observable in vitro and in vivo [34,35,36]. Recently, a synthetic triterpenoid NRF2 modulator and bardoxolone-derivative, RTA 408, has been tested for topical NRF2 activation in rat, murine, and human skin [37,38], but limited data on skin protection properties are available. Taken together, a significant opportunity for the development of cutaneous NRF2-dependent skin protection strategies using nutrient-derived molecular entities remains to be explored.

## 4. NRF2 Control of Skin Barrier Structure and Function

Recently, it has been shown that numerous genes encoding skin barrier structural and functional components are under NRF2 transcriptional control, including late cornified envelope 1 (LCE1) family members (*LCE1B*, *LCE1C*, *LCE1E*, *LCE1G*, *LCE1H*, *LCE1M*), keratins (*KRT6A*, *KRT16*, *KRT17*), small proline rich proteins (*SPRR2D*, *SPRR2H*), secretory leukocyte protease inhibitor (*SLPI*), and the EGF family member epigen (*EPGN*), some of which contain a validated ARE [39,40,41,42,43]. Moreover, a novel role of NRF2 in skin barrier and desmosome function has been attributed to transcriptional control of MiR-encoding genes (*MIR29AB1* and MIR29B2C) in keratinocytes, substantiating a novel NRF2-miR29-DSC2 (desmocollin-2) axis in control of desmosome function and cutaneous homeostasis [44]. In addition, much research has substantiated a role of NRF2 in epidermal redox control, stress response regulation, terminal differentiation, and barrier homeostasis, and a crucial role of NRF2 in the control of a cytoprotective glutathione gradient throughout the epidermis has been demonstrated [13,35,40,41,45].

Additional functional implications of NRF2 relevant to skin barrier maintenance, repair, and rejuvenation have recently emerged, including a role in metabolic control and mitochondrial homeostasis, proteasomal function and autophagy, and stem cell renewal and pluripotency [46,47,48].

Moreover, abundant functional crosstalk exists between NRF2 and other cutaneous stress response pathways including AhR (arylhydrocarbon receptor) and NFκB [49,50,51]. For example, the co-occurrence of ARE- and xenobiotic response element- (XRE-)sequences in the promoter region of several AhR-controlled genes (including NQO1 (NAD(P)H quinone oxidoreductase 1) and GST (glutathione-S-transferase) indicates mechanistic crosstalk between NRF2 and AhR at the gene expression level [52]. Likewise, direct AhR binding to XREs located in the NRF2 promoter region has been confirmed by immunoprecipitation analysis, enabling AhR agonists to induce NRF2 expression at the mRNA and protein levels. It has also been demonstrated that protease-activated receptor-2 (PAR-2), an important mediator of inflammation and immune responses by serine proteinases, activates NQO1 via NRF2 stabilization in keratinocytes, suggesting that in addition to induction of inflammation, PAR-2 can play a cytoprotective role that depends on NRF2 [53].

## 5. NRF2 in Skin Pathology

A substantial body of experimental evidence indicates that NRF2 dysregulation, either due to insufficient adaptive activation in response to environmental stressors or due to constitutive hyperactivation as a result of genetic alterations that may also involve KEAP1, has detrimental effects compromising skin barrier function and stress responses. Seminal research has documented that constitutive epidermal NRF2 overactivation through permanent genetic deletion of KEAP1-caused hyperkeratosis in murine skin [54]. It has also been demonstrated that forced constitutive NRF2 overactivation causes chloracne-like skin disease characterized by acanthosis, hyperkeratosis, and cyst formation in mice [43]. Likewise, oncogenic NRF2 mutations have been detected in squamous cell carcinomas of the esophagus and skin [55,56,57]. In contrast to compromised skin structure and function that may originate from both impaired NRF2 activation as well as forced hyperactivation, NRF2 activation in healthy skin is transient and subject to extensive feedback regulation and modulatory crosstalk. Pharmacological modulation of NRF2 in skin aiming at a therapeutic, preventive, or regenerative benefit must therefore be performed without causing prolonged hyperactivation of the pathway as has been discussed before [56,58].

Wound healing. Recent research indicates that a glutathione-NRF2-thioredoxin cross-talk enables keratinocyte survival and wound repair through modulation of inflammation, apoptosis, and oxidative stress [59]. Importantly, substantial research has identified an essential role of NRF2 in diabetic wound healing, amenable to therapeutic intervention using small molecule NRF2 activators such as sulforaphane and cinnamaldehyde [32,60].

Psoriasis. In psoriasis, NRF2 is an important driver of keratinocyte proliferation with up-regulation of Keratin 6, Keratin 16, and Keratin 17 [61]. However, NRF2-directed intervention in psoriasis is efficacious since the anti-psoriatic drug monomethylfumarate increases NRF2 levels and induces aquaporin-3 mRNA and protein expression, important for keratinocyte differentiation [62].

Allergic dermatitis. NRF2 activation has been identified as a key event triggered by common skin sensitizers known be cysteine-directed electrophiles [63,64,65,66]. However, pharmacological NRF2 activation using ginger-derived 6-shogaol has shown efficacy in allergic dermatitis-like skin lesions through anti-inflammatory redox modulation [67].

Atopic dermatitis. Redox dysregulation is an emerging causative factor contributing to compromised skin barrier function in atopic dermatitis, and pharmacological intervention targeting NRF2 has shown promise targeting atopic dermatitis-like skin lesions in 2,4-dinitrochlorobenzene (DNCB)-sensitized and challenged mice [68,69].

Melanocytic dysfunction. It is now understood that NRF2 also plays an essential role in the maintenance of melanocyte responses to environmental stressors. NRF2 has been implicated in cutaneous pigmentation disorders resulting from redox alterations relevant to vitiligo and stress-induced and chronological hair greying [70,71,72,73]. Interestingly, recent evidence suggests that NRF2 plays a role in facilitating glutathione-dependent chemoresistance of malignant melanoma cells [74].

Chronological aging and progeria. Increasing evidence indicates a role of NRF2 in the control of chronological cellular aging [75,76,77]. Recently, an unanticipated mechanistic role of NRF2 dysfunction as a key contributor to premature aging has been proposed in the genetic premature aging disorder Hutchinson-Gilford progeria syndrome (HGPS), attributed to increased chronic oxidative stress [78,79]. In HGPS, a de novo LMNA (lamin A/C) gene mutation encodes for progerin, a dysfunctional nuclear architectural protein variant of lamin A lacking 50 amino acids. Progerin formation is also observed during normal cellular aging, and chronic UVA exposure has been shown to induce progerin in cultured human dermal fibroblasts [80]. Recent experimental evidence suggests that progerin sequesters NRF2 and thereby causes its subnuclear mislocalization, resulting in impaired NRF2 transcriptional activity and consequently increased chronic oxidative stress. Importantly, reactivation of NRF2 activity in HGPS patient cells reverses progerin-associated nuclear aging defects, suggesting that progerin-dependent repression of NRF2-mediated antioxidant responses is a key factor underlying HGPS-type premature aging with potential relevance to chronological aging and UVA-induced photoaging.

NRF2 in skin photodamage. Recent studies strongly suggest a protective role of NRF2-mediated gene expression in the suppression of cutaneous photodamage induced by solar UV radiation (as evidenced by suppression of UV-induced apoptosis and inflammatory signaling), and NRF2 activation has been shown to protect cutaneous keratinocytes and fibroblasts against the cytotoxic effects of UVA and UVB [16,18,19,31,33,81,82,83,84,85,86,87,88]. Importantly, research performed in SKH-1 mice documents that genetic NRF2 activation protects mice against acute photodamage and photocarcinogenesis [36,89]. Therefore, pharmacological modulation of NRF2 has now attracted considerable attention as a novel approach to skin photoprotection, cancer photochemoprevention, and suppression of skin photoaging [13,33,34,86]. Indeed, protection of primary human keratinocytes from UVB-induced cell death by novel drug-like NRF2 activators has been reported, a photoprotective effect attributed in part to NRF2-dependent elevation of cellular glutathione levels [40,87,90]. 

Our own studies have demonstrated the photoprotective effects of pharmacological NRF2 activation in cultured human skin cells and reconstructed epidermal skin models [13,31,33]. Topical application of NRF2 inducers, e.g., the synthetic NRF2-activator TBE-31, has shown pronounced photoprotective and photochemopreventive activity in murine skin, and suppression of solar UV-induced human skin erythema was achieved by topical application of a standardized broccoli extract delivering the NRF2 inducer sulforaphane [36]. However, little research has explored the concept of cutaneous photoprotection and photochemoprevention achievable by systemic administration of NRF2 inducers [13,91].

## 6. Systemic Photoprotection by Dietary NRF2 Activators: Focus on the Apocarotenoid Bixin, an FDA-Approved Food Colorant and Spice Native to Tropical America

**Figure 2:**
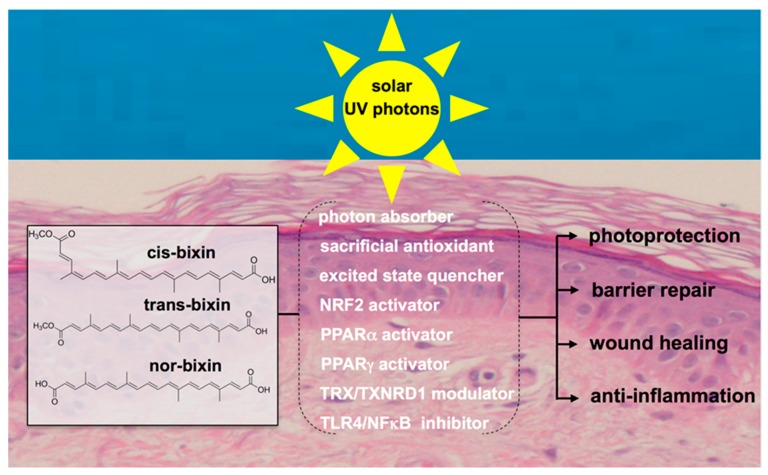
Bixin for improved skin barrier function and photoprotection. Based on pleiotropic activities including direct chemical and NRF2-dependent antioxidant modulation, cis-bixin and its physiologically relevant derivatives trans-bixin and nor-bixin enhance skin barrier structure and function with photoprotective and potentially photochemopreventive efficacy; thioredoxin (TRX), thioredoxin reductase 1 (TXNRD1).

The dietary origin of numerous photochemopreventive factors suggests the possibility of achieving efficient skin delivery through oral systemic administration, an emerging concept referred to as ‘nutritional’ or ‘systemic photoprotection’ [9]. Indeed, clinical studies document feasibility of human skin photoprotection by dietary intake of lycopene from processed tomato and flavonoid-rich cocoa [10,92,93,94]. In an attempt to test for the first time the feasibility of NRF2-dependent systemic photoprotection by dietary constituents, we focused our photoprotection studies on the apocarotenoid bixin (Figure 1 and Figure 2), an FDA-approved natural food colorant from the seeds of the achiote tree (*Bixa orellana*) native to tropical America [13,95,96]. A native spice derived from the Americas, annatto is an orange-red condiment and food coloring used to impart a yellow or orange color to signature foods of Latin America and the Caribbean.

Consumed by human populations in the Americas since ancient times, this apocarotenoid, derived from lycopene through oxidative cleavage, is now used worldwide as a spice, food colorant, and cosmetic and pharmaceutical ingredient (referred to as ‘annatto’; E160b). Due to its unusual (linear/noncyclic) chemical structure, the apocarotenoid bixin displays characteristics different from all other carotenoids. Specifically, bixin is water soluble, does not display provitamin A activity, and is distinguished by an excellent safety record as well as established systemic bioavailability and pharmacokinetic profile upon oral administration as documented extensively in mice and humans [97,98,99]. Indeed, bixin is now one of the most consumed food colorants in the world distinguished by a long record of dietary and ethno-pharmacological use [95,96,100]. Chemical activities of bixin as sacrificial antioxidant, free radical scavenger, and efficient physical quencher of photoexcited states including singlet oxygen (surpassed only by lycopene) are documented [101]. Topical preparations of annatto extract have been in ethno-pharmacological use showing therapeutic efficacy for wound healing, mouth ulcers, and other pathologies associated with impaired epithelial barrier function [100,102]. It is also interesting that translational research documents the efficacy of bixin-loaded polycaprolactone nanofibers as an innovative delivery system accelerating wound healing and reducing scar tissue formation in diabetic mice [103]. Moreover, bixin-based systemic protection against environmental toxicants including methylmercury and carbon tetrachloride has been documented in vivo [104,105]. 

In prior studies, bixin has demonstrated antigenotoxic and antioxidant cytoprotective activities, and systemic availability of oral bixin and its demethylated metabolite norbixin has been documented in rodent studies and healthy human subjects [97,98,106,107]. In long term murine feeding experiments, supplementation levels up to 5% (*w*/*w* food) were well tolerated. Importantly, acceptable daily intake (ADI) over a lifetime without an appreciable health risk (http://apps.who.int/food-additives-contaminants-jecfa-database/search.aspx) surpasses that of any other carotenoid approved as a food additive [ADI (bixin): 12 mg/kg body weight/day] [108]. 

## 7. Bixin for NRF2-Dependent Systemic Skin Photoprotection

Bixin was identified as the result of a screen for diet-derived small molecule NRF2 activators targeting oxidative stress and redox dysregulation in epithelial cells [13,109]. Using activity guided fractionation and bio-analytical tools for the quantitative detection of bixin and other small molecule constituents in annatto extracts, we were able to demonstrate that bixin is the active molecular entity in annatto total organic extracts responsible for NRF2 activation. Recently, we have reported for the first time that (i) bixin is a potent activator of the NRF2-dependent cytoprotective response in cultured human skin keratinocytes; (ii) systemic administration of bixin activates cutaneous NRF2 with potent protective effects against solar UV-induced skin damage in SKH-1 mice; and (iii) bixin-induced suppression of photodamage is observable in *Nrf2*^+/+^ but not in *Nrf2*^−/−^ SKH-1 mice confirming the NRF2-dependence of bixin-based antioxidant and anti-inflammatory cutaneous effects [13]. Based on its unique status as a FDA-approved food additive with an established safety profile and potent NRF2-inducing activity, we also have investigated and established efficacy of systemic NRF2 activation using intraperitoneal administration of bixin for lung protection against ventilation-induced oxidative stress [110]. Importantly, dietary carotenoids (including β-carotene, lycopene, lutein, 3,3′-dihydroxyisorenieratene, zeaxanthin, astaxanthin) and their biosynthetic precursor molecules (such as phytoene) have been under investigation for epithelial chemoprevention and cutaneous photoprotection before [10,92,111,112,113], and the systemic photoprotective activity of carotenoids, displayed only after dietary uptake and cutaneous accumulation, has largely been attributed to their activity as photon absorbers, sacrificial antioxidants, and excited state/singlet oxygen quenchers [101,113,114].

Interestingly, it has been shown that astaxanthin and its analogs (such as adonixanthin) activate NRF2, preventing light-induced ocular photoreceptor degeneration [115]. Moreover, fucoxanthin, another marine carotenoid from seaweed, has been shown to enhance the level of reduced glutathione via NRF2 in human keratinocytes [116]. Indeed, prior research has examined the specific mechanism of NRF2 activation by carotenoids, and oxidative metabolism leading to the generation of electrophilic unsaturated mono- and dialdehydes (such 10,10′-diapocarotene-10,10′-dial) has been identified as the mechanistic basis underlying upregulated antioxidant responses [117,118,119]. The specific structure-activity relationship of NRF2 upregulation by carotenoid-derived electrophilic metabolites has been explored before, and it is therefore likely that bixin-dependent NRF2 activation requires similar oxidative transformation to electrophilic intermediates, a subject of ongoing investigation. However, even though the concept of cutaneous photoprotection achieved by systemic administration of specific carotenoids and other phytochemicals has been explored in the past [10,92,111,112,120,121,122], prior to our own investigations, no research had investigated the NRF2-dependence of carotenoid-based systemic photoprotection [13]. However, the biological effects of prolonged cutaneous NRF2 activation as a consequence of oral/systemic delivery of a pharmacological molecular agent that may also affect NRF2 regulation in non-cutaneous tissue remain to be elucidated. 

## 8. Other Molecular Targets of Bixin with Relevance to Skin Barrier Function and Protection

Beyond NRF2-directed activities, bixin has been demonstrated to cause specific modulation of the following molecular targets potentially relevant to skin barrier function and environmental stress responses (Figure 2).

### 8.1. PPARα and PPARγ

Interestingly, peroxisome proliferator-activated receptors (PPARs) have now been recognized as important determinants of keratinocyte responses to skin injury regulating skin homeostasis, epithelial repair, and morphogenesis [123,124]. Specifically, PPARα is a ligand-activated transcription factor that regulates the expression of genes involved in fatty acid oxidation.

Recently, it has been demonstrated that oral administration of bixin improves obesity-induced abnormalities of carbohydrate and lipid metabolism in mice, an affect attributed to PPARα activation confirmed by luciferase reporter assays [125]. Specifically, treatment with bixin- and norbixin-induced PPARα target gene expression upstream of fatty acid oxidation in PPARα-expressing HepG2 hepatocytes. Likewise, in obese KK-Ay mice, chronic nutritional supplementation using bixin suppressed the development of hyperlipidemia and hepatic lipid accumulation with improvement of hyperglycemia, hyperinsulinemia, and hypoadiponectinemia. This effect is consistent with upregulated mRNA expression levels of adiponectin (*ADIPOQ*), an adipocyte-derived adipokine with multiple beneficial effects such as anti-obesity and anti-insulin resistance roles as well as anti-apoptotic, anti-oxidative, and anti-inflammatory activities in skin [124]. Likewise, experimental evidence suggests that bixin also enhances adipocyte insulin sensitivity downstream of PPARγ activation [126]. It is therefore tempting to speculate that the documented beneficial effects of bixin on cutaneous barrier function and wound healing may be in part attributable to PPARα/γ-directed agonism operative in addition to NRF2 activation as discussed above. However, the effects of prolonged pharmacological PPARα- or γ-directed agonism on skin barrier function remain to be explored. 

### 8.2. Thioredoxin/Thioredoxin Reductase

One of the key cellular antioxidant systems is regulated by the selenoproteins thioredoxin (TRX) and thioredoxin reductase (TXNRD1), which use NADPH as an electron donor to reduce oxidized substrates. TXNRD1 contains a very reactive selenocysteine in its active site that is prone to electrophilic or oxidative attack, making it another important sensor of the cellular redox state in addition to KEAP1 [127]. Thus, electrophilic compounds that typically activate NRF2 by KEAP1 cysteine modifications will also inhibit TXNRD1 [127]. The TRX/TXNRD1 system is essential for keratinocyte survival, UV protection, and wound healing [59]. Interestingly, one report indicates that at high (200 μM) concentrations bixin generates ROS, inhibiting both TRX and TXNRD1 with induction of cell death [128]. This could be due to an exacerbated redox imbalance caused by the inability of TRX/TXNRD1 to reduce their substrates, such as peroxiredoxins (PRX), as well as de-repression of proapoptotic proteins, such as apoptosis signaling kinase 1 (ASK1), apoptosis inducing factor (AIF), and caspase 3. Other important substrates of the TRX/TXNRD1 system are PTEN (phosphatase and tensin homolog), NF-κB, AP1 (activator protein 1), and p53 (tumor protein 53), with important implications for regulation of cell survival in response to TRX/TXNRD1 dysruption [129]. Interestingly, it has been proposed that the TRX/TXNRD1 system might reduce the oxidized cysteine residues in KEAP1 to restore its functionality [130]. Dual inactivation of these reactive proteins (KEAP1 and TRX/TXNRD1) could contribute to pronounced NRF2 activation achieved by bixin. However, since TRX and TXNRD1 are NRF2 target genes, reduced proteins might be restored by de novo synthesis and GSH synthesis.

### 8.3. TLR4/NFκB

It has been observed that nutrional bixin attenuates cardiac injury progression through inhibition of fibrosis, inflammation, and redox dysregulation, cytoprotective effects that were attributed to Toll-like receptor 4/nuclear factor kappa B (TLR4/NF-κB) antagonism in mice [131]. Likewise, bixin antagonized lipopolysaccharide (LPS)-induced pro-inflammatory cytokine over-expression in cultured cardiac muscle cells. Given the emerging importance of TLR4 signaling in skin inflammation and UV-induced photodamage, it is therefore tempting to speculate that nutritional bixin regimens may benefit human skin through TLR4 antagonism operative in addition to NRF2 activation [132,133].

## 9. Conclusions

The promising concept of achieving cutaneous solar protection through dietary intake of NRF2 activators remains largely unexplored, representing an innovative molecular strategy that deserves further exploration. Building on its excellent safety record as an FDA-approved natural food colorant and additive, its systemic availability upon oral administration in humans, and ability to activate NRF2 in skin, dietary consumption of bixin, an ethno-pharmacologically validated phytochemical originating from the Americas, warrants future preclinical and clinical evaluation for improved skin barrier function and photoprotection.
